# HIV and Comorbidities – the importance of Gut Inflammation and the Kynurenine Pathway

**DOI:** 10.1097/COH.0000000000000782

**Published:** 2022-12-28

**Authors:** R MacCann, A.L. Landay, Paddy W.G. Mallon

**Affiliations:** a UCD Centre for Experimental Pathogen Host Research (CEPHR), School of Medicine, University College Dublin, Dublin 4; b St Vincents University Hospital, Elm Park, Dublin 4, Ireland; c Department of Internal Medicine, Rush University Medical Center, Chicago, Illinois, USA

**Keywords:** HIV, microbiome, microbiota, dysbiosis, kynurenine, geroscience, senotherapeutics

## Abstract

**Purpose of Review:**

The purpose of this article is to review alterations in microbiota composition, diversity, and functional features in the context of chronic inflammation and comorbidities associated with HIV infection.

**Recent Findings:**

The gut microbiome is an important mediator of host immunity, and disruption of gut homeostasis can contribute to both systemic inflammation and immune activation. Ageing and HIV share features of intestinal damage, microbial translocation and alterations in bacterial composition that contribute to a proinflammatory state and development of age-related comorbidities. One such inflammatory pathway reviewed is the NAD+ producing Kynurenine Pathway (KP). Kynurenine metabolites regulate many biological processes including host-microbiome communication, immunity and oxidative stress and the KP in turn is influenced by the microbiome environment. Age-associated decline in NAD+ is implicated as a driving factor in many age-associated diseases, including those seen in PWH. Recent studies have shown that KP can influence metabolic changes in people with HIV (PWH), including increased abdominal adiposity and cardiovascular disease (CVD). Furthermore, KP activity increases with age in the general population, but it is elevated in PWH at all ages compared to age-matched controls. Host or microbiome-mediated targeting of this pathway has merits to increase healthy longevity and has potential therapeutic applications in PWH.

**Summary:**

As a growing proportion of PWH age, many face increased risks of developing age-related comorbidities. Chronic inflammation, a pillar of geroscience, the science of ageing and of age-related disease, is influenced by the gut microbiome and its metabolites. Combined, these contribute to a systemic inflammatory signature. Advances in geroscience-based approaches and therapeutics offer a novel paradigm for addressing age-related diseases and chronic inflammation in HIV infection. Whether targeted inhibition of KP activity alleviates pathological conditions or promotes successful ageing in PWH remains to be determined.

## Introduction

Since the introduction of combination antiretroviral therapy (ART), PWH are now achieving the life expectancy of the general population (1). Evidence suggests that treated HIV infection is associated with development of age-related complications at a higher rate, and in some cases at an earlier age than the HIV-negative population (2). The disproportionately greater prevalence of ageing-related comorbidities in PWH extends across both resource-rich and limited care settings (3). This has led to the application of the geroscience hypothesis to PWH, which proposes that the root cause of most ageing-related chronic diseases and conditions is the ageing process itself. Multiple components, including environmental, socioeconomic, psychological as well as biological factors, contribute to ageing. One significant contributor in relation to chronic disease is unresolved inflammation and chronic immune activation (4). Indeed, late-life, chronic, low-grade inflammation and altered signal transduction pathways in metabolism have been identified as two key pillars of geroscience. One such signal transduction pathway is Tryptophan (TRP) metabolism through the kynurenine pathway (KP), which plays essential roles in energy production and ageing.

The gut microbiome is an important mediator of host immunity, and disruption of gut homeostasis can result in systemic inflammation and immune activation (5). HIV infection is associated with alterations in microbiota composition, microbial metabolites, disruption of the gut endothelial barrier, and the production of microbial toxins such as lipopolysaccharide (LPS) (6). These changes may contribute to greater microbial translocation and the persistence of a pro-inflammatory state even after restoration of circulating CD4+ T cell counts under successful ART(5). Combined with chronic innate immune activation, these processes play a role in the development of comorbid conditions in PWH (7,8). The study and management of these comorbid conditions has focused on the unique signatures related to each condition. Yet, extending from the geroscience hypothesis, therapeutically targeting fundamental aging processes along a common pathway of inflammation could have a greater impact on alleviating or delaying aging-associated comorbidities than addressing each disease individually. The use of pharmacological agents, or “Senotherapeutics” to combat age-related diseases, have been proposed as a Geroscience treatment approach.

In this review, we aim to focus on recent publications about microbial translocation, the kynurenine pathway, and the development of comorbidities in PWH. Furthermore, we will discuss how the geroscience theory could be applied in the management of these comorbidities and the role senotherapeutics could have in PWH. Such an understanding can inform prevention strategies, management, and subsequent research directions.

## HIV and microbiome compositional changes

HIV infection is associated with a disruption of microbial composition, decreased microbial diversity and shifts in composition, including increases in pathogenic and decreases in beneficial gut microbial species. Some of these compositional patterns are unique to PWH, with enrichment of potential pathobioints *Fusobacteria*, *Enterobacteriaceae*, *Prevotella*, and *Proteobacteria* species, and depletion of beneficial short-chain fatty acid-producing bacteria that are anti-inflammatory such as *Bacteroides*, *Ruminococcus*, *Lactobacillus*, and *Bifidobacterium* species (9). These compositional changes are associated with an overall increase in inflammation, systemic immune activation, and microbial translocation in PWH that have been linked to HIV disease progression and the development of non-AIDS-related comorbidities (8)(9)(10).

For instance, gut microbial compositional changes are seen in PWH with cardio-metabolic comorbidities, with decreased abundance of *Bifidobacterium*, *Bacteroides* and *Akkermansia* seen in PWH with type 2 diabetes (T2DM)(10). These changes in composition extend even to those with prediabetes as described in a recent cross-sectional analysis of 40 PWH, 20 of whom had prediabetes and 20 who were normoglycaemic. This study found that alpha-diversity, or the measure of microbiome diversity applicable to a single sample, and beta-diversity, a measure of the similarity or dissimilarity of two communities, was significantly lower in PWH with prediabetes than in those with normoglycemia and that relative abundance of two genera in *Firmicutes* (*Streptococcus* and *Anaerostignum*) were significantly higher in the prediabetes group (11).

In PWH and the general population, there is an overall reduction in gut microbial diversity in obese compared to lean participants, with higher levels of microbes from phylum *Firmicutes* and lower proportions of *Bacteroidetes* (12,13). This relative abundance of *Firmicutes* could be responsible for an increase in the capacity to digest polysaccharides, giving rise to increases in monosaccharides and short-chain fatty acids (SCFA) capable of being absorbed by the host, thus leading to increased caloric intake and weight gain. In PWH, a two-fold risk of metabolic syndrome was found in the microbiome profiles that had a high HIV-related microbiota index, mainly driven by enrichment of *Desulfovibrionaceae*, hydrogen sulfide-producing bacteria which can cause toxic effects, and decrease in several *Clostridia* species, known butyrate-producers (14).

## Gut microbiome and Butyrate

Microbial metabolites, byproducts produced by intestinal bacteria, are also important in influencing immune responses(15). Butyrate is one of the SCFAs produced as end-products of intestinal microbial fermentation. Butyrate plays an important role in immune regulation through several mechanisms. It can support the integrity of the intestinal epithelial barrier by regulating the expression of tight junctional proteins, it assists with gut motility and has anti-inflammatory properties by downregulation of the NF-κB signalling pathway which is involved in gene transcription of inflammatory cytokines (16). Butyrate production also correlates with a decrease in monocyte activation and inflammation as measured by circulating soluble CD14 (sCD14) and C-reactive protein (CRP)(17). Lower levels of butyrate-producing bacteria (BPB) are associated with inflammation and insulin resistance (18). In PWH, decreased abundance of BPB is seen in untreated PWH compared to HIV-uninfected subjects and this was correlated with microbial translocation, immune activation and vascular inflammation (19). Furthermore, lower BPB in those with T2DM, metabolic syndrome and dyslipidaemia is seen in the general population as well as PWH (12,20,21).

## HIV, microbial translocation and clinical comorbidities

PWH have increased risk of developing cardiovascular disease (CVD) and metabolic disease beyond that explained by traditional risk factors. CVD is now the leading cause of death in PWH on ART and PWH have an approximate two-fold increased risk of myocardial infarction(22). Furthermore, compared to the general population, prediabetes and T2DM in PLWH are more prevalent(23). Chronic systemic inflammation resulting in dysregulation of glucose and lipid trafficking, utilization, storage, alongside elevations in biomarkers of inflammation are thought to contribute to increased CVD and metabolic risk in PWH. The gut microbiome may additionally impact metabolic syndrome and CVD through effects on intestinal barrier function, leading to microbial translocation and systemic inflammation(24). Microbial markers, such as LPS and sCD14 have been linked to innate immune activation, atherosclerosis, coronary artery calcification and clinical vascular disease in PWH on ART(25–27).

Fungal mass constitutes the second player after bacterial mass in the composition of gut microbiota and so markers of fungal translocation are also associated with immune activation and systemic inflammation in PWH. Intestinal fatty-acid binding protein (I-FABP) levels, a marker of enterocyte damage, are consistently higher in people chronically infected with HIV compared to HIV-negative controls(28) and higher circulating I-FABP is associated with increased mortality and lower CD4+ T-cell counts(29). The role of fungal translocation on metabolic alterations and inflammation in HIV is beginning to be realised. I-FABP was found to be inversely related to BMI and adiposity in chronic HIV infection(28) and Beta-D-Glucan (BDG), a marker of fungal translocation, was associated with increased adiposity over 96 weeks following ART initiation. This association remained when accounting for confounders such as age, physical activity, smoking, alcohol and drug history, race, and sex. (30). Following this, a study in 2021 showed an association between plasma BDG levels and subclinical coronary atherosclerosis plaque in 93 ART-treated PLWH but not uninfected controls(31). All participants included were over the age of 40 and had a 10-year Framingham risk score ranging from 5% to 20%. It is interesting to note that the commonly used marker of microbial translocation LPS was not elevated in either of these BDG studies, however, LPS is not always a reliable marker of bacterial translocation given its short half-life and interference by other factors. Thus, although BDG appears as a new marker associated with non-AIDS comorbidities, current observations rely on associations only. More studies are thus needed to determine the mechanism linking fungal translocation and comorbidities.

Taken together, these changes in microbiome composition and function in PWH contribute to a chronic inflammatory state in some PWH that persists even after restoration of circulating CD4+ T cell counts under successful ART(5). Moreover, ageing and HIV share these features of intestinal damage and alterations in bacterial composition, each contributing to this pro-inflammatory state and development of age-related comorbidities(32). These shared characteristics could be targeted as a therapeutic strategy, as introduced by the geroscience theory, which proposes to target the ageing process itself. One interesting target worth discussing is the kynurenine (KP) metabolic pathway.

## Kynurenine pathway and age-related comorbidities in HIV

**Figure 1:**
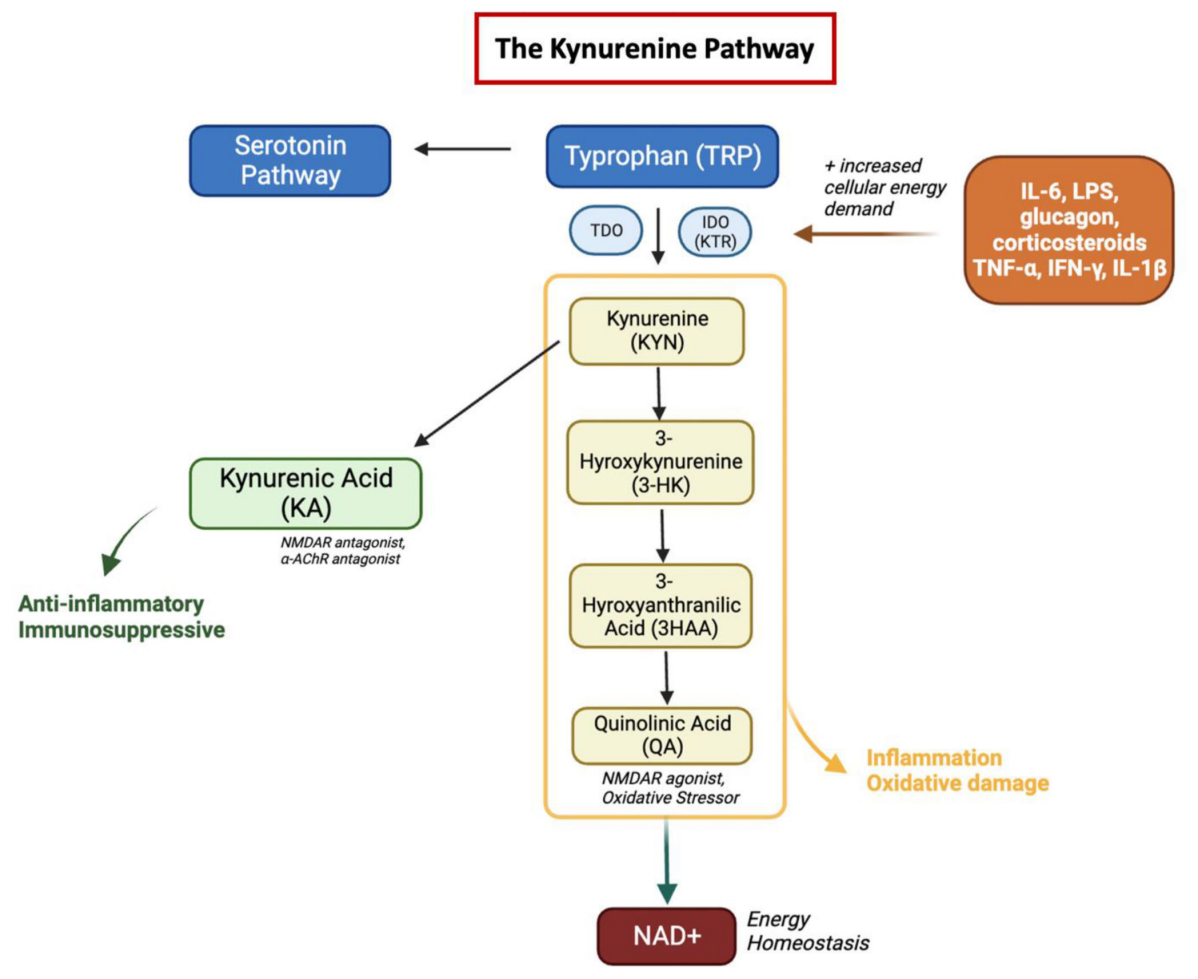
The Kynurenine Pathway. In the kynurenine pathway, TRP is metabolized to kynurenine (KYN) by tryptophan 2,3-dioxygenase (TDO), found mostly in hepatic tissue or indoleamine 2,3-dioxygenase (IDO), an enzyme found in most tissues that is stimulated by steroid hormones, cytokines and growth factors. Kynurenine is then either metabolized to kynurenic acid (KA) or to 3-hydroxykynurenine (3-HK). Under basal conditions, most of kynurenine is metabolized to KA, a N-methyl-D-aspartate (NMDA) and α7-nicotinic acetylcholine (α7nACh) receptor antagonist. However, inflammation, oxidative stress and pro-inflammatory cytokines will shift kynurenine metabolism to 3-HK. Further metabolism through this pathway results in quinolinic acid (QA), a metabolite that is a NMDA receptor agonist and an oxidative stressor. The KP produces several other biologically active metabolites, including the redox cofactors oxidized NAD+. NAD is a common mediator of various biological processes, including energy metabolism, mitochondrial function, calcium homeostasis and generation of oxidative stress. TRP metabolites like serotonin, quinolinic acid, and kynurenic acid have important implications in neurotransmission, growth and inflammation. IDO, indoleamine 2,3-dioxygenase; NAD, nicotinamide adenine dinucleotide.

The KP is the sole *de novo* nicotinamide adenine dinucleotide (NAD+) biosynthetic pathway, generating NAD+ from ingested tryptophan (TRP)(33) (See Figure 1). NAD+ is an essential cofactor that plays a critical role in energy production as well as many enzymatic redox reactions. The first and rate-limiting step of the kynurenine pathway is made by tryptophan 2,3-dioxygenase (TDO) or indoleamine 2,3-dioxygenase (IDO), the activity of which can be measured using the kynurenine/ tryptophan ratio (KTR) as a surrogate marker. IDO activity levels, measured by KTR, increase with age in a general population cohort without known infections and is reflected by an age-associated decline in NAD+ levels and energy production (34). This age-associated decline in NAD+ is implicated as a driving factor in many age-associated conditions, including metabolic and neurodegenerative disease (35–37).

Gut bacteria can alter the rate and availability at which tryptophan metabolism occurs through the KP and this can both protect the host from excess tryptophan, as well as produce beneficial kynurenine metabolites. Thus, the KP is a double-edged sword; it has key roles in cell growth, cell maintenance and neurotransmission but can also drive inflammation and systemic immune responses. Since many microbial organisms rely on the essential amino acid TRP, its’ degradation through IDO/TDO activity induced by exogenous pathogens can limit infection through TRP starvation. Thus, by reducing available substrate for microorganisms, the KP acts to inhibit growth during acute infections(33). This pathway constitutes a delicate balance between pathogen defense and host protection and is influenced by various stressors including infection, inflammation, and oxidative stress. Altered levels of KP metabolites, such as kynurenic acid (KA) or quinolinic acid (QA) have been reported in numerous disease processes, including metabolic syndrome (38), CVD(39), obesity (40), frailty, and neurologic diseases such as mental health disorders (36). Indeed Kynurenic acid (KA) can serve as an early biomarker for diabetes and some other metabolic diseases (41). Given these diverse physiological functions, metabolites of the KP are emerging as key targets in diseases such as diabetes, atherosclerosis, and more recently in HIV (42).

The link between KP and HIV infection has been known since 1998 when increased KTR was seen in PWH thus suggesting the link between increased KP activity and HIV immune dysfunction (43). The KTR represents an as independent marker of CD4 T-cell counts, with a higher KTR associated with lower CD4 T-cell counts and more advanced stages of disease (44). Furthermore, microbiota alterations in PWH have been linked with tryptophan catabolism and systemic inflammation (45). Increased KTR corresponds with detectable levels of microbial translocation from the gastrointestinal tract and gut microbiota changes showing a dysbiotic mucosal-adherent community enriched in *Proteobacteria* and depletion of *Bacteroidia*. Despite early initiation of ART, we see little improvement in this mucosal dysfunction or KP activity in PWH (46), enabling a self-sustaining feedback loop of inflammation. This constitutes a vicious cycle of immune activation, immune tolerance, senescence, and exhaustion. This immunophenotype of PWH and KP metabolism is similar to that seen in older people.

The KP has been closely linked with HIV-specific comorbidities such as HIV-associated neurogenerative disease (HAND) as well as both CVD and T2DM in PWH (47,48,49). Elucidating this link between inflammation in PWH and metabolic clinical outcomes has been an expanding area of research. The Copenhagen Comorbidity in HIV infection (COCOMO) study is an ongoing, longitudinal, observational study with the aim of assessing the burden of non-AIDS comorbidities in PWH. In a 2019 study they looked at 864 PWH and 75 uninfected controls to assess possible associations between KP metabolites and serum lipids, hypertension, and diabetes. They found that an increase in abdominal adipose tissue was associated with increased KTR in PWH (42). In 2022 they went one step further, and a new analysis of the COCOMO cohort that included 383 PWH who were mostly male, virologically suppressed on stable ART and showed that HIV-related gut microbiota alterations were associated with increased KTR. This in turn was associated with indices of visceral abdominal tissues (VAT)(50).

These patterns of inflammation induced by the KP in PWH share features with those seen in ageing (51). A recent retrospective multi-site cohort study examined the relationship of KTR, bacterial translocation, and ageing in 205 PWH on ART that had a median age of 52 years. They showed that although KTR increases with age in the general population, it is elevated in PWH at all ages compared to age-matched controls(52). This study provides an accurate reflection of the impact age has on KP activity in PWH, as they excluded subjects with comorbidities such as malignancy, hepatitis, and autoimmune disease as well as those on immunosuppressive medication. Unlike previous studies, they did not appreciate a relationship between KP activity and microbial translocation, but they only used LPS as an isolated marker of bacterial translocation. These findings suggest that a disturbed gut microbiota composition and tryptophan catabolism could represent pathogenic pathways potentially interacting in HIV infection and age-related comorbidities.

## The Geroscience approach to gut microbiome modification in HIV

Efforts to target drivers of chronic inflammation in PWH through microbiome modification have been met with modest success. Interventions targeting microbial translocation with the use of probiotics, prebiotics and when combined in the form of synbiotics, have had varying results (17,53). Other studies have tried to target microbial translocation in PWH by reducing circulating LPS or sCD14 levels with the use of drugs such as sevelamer (54) and rifaxmin (55), but these treatment strategies failed to reduce levels of markers of inflammation. Clinical trials assessing the clinical outcomes of fecal transplantation in PWH have had limited results, but suggest good tolerability and feasibility (56).

Recently, attention has turned to the geroscience hypothesis, which proposes that therapeutically targeting fundamental mechanisms that underly ageing will have a significantly greater impact on overall human disease burden than an individual disease treatment approach. Hallmarks of ageing include inflammation, impaired adaptation to stress, mitochondrial dysfunction, altered metabolism and immunosenescence. These processes have been viewed historically as distinct types of biological processes, but evidence suggests that these pillars of ageing are intricately linked (57).

**Figure 2:**
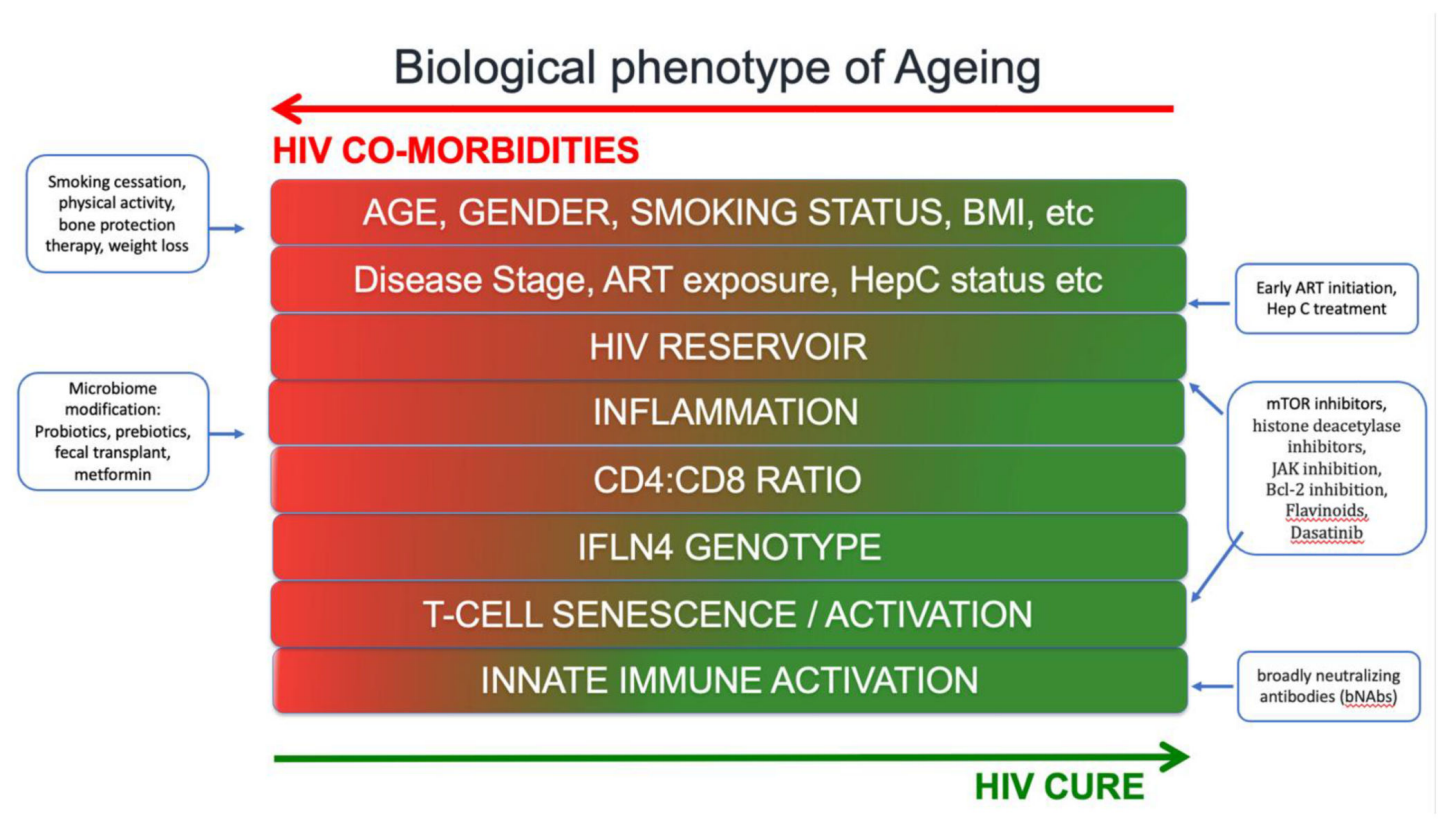
Biological phenotype of ageing and therapeutic modification strategies.

The use of pharmacological agents to combat age-related diseases, termed senotherapeutics, have been proposed as a geroscience treatment approach. These pharmacologic agents act either by selectively promoting the death of senescent cells (‘senolytics’) or modifying senescent phenotype (‘senomorphics’)(58). Cellular senescence is an age-related phenotype, related to HIV disease progression, ART and comorbidities in PWH (59). Several senolytics have been explored as part of investigational HIV cure strategies by targeting the HIV viral reservoir and include the flavinoid Quercetin, the BCL-2 antagonist Venetoclax, the mTOR inhibitor Rapamycin, the JAK 1/2 inhibitor Ruxolitinib and the histone deacetylase inhibitor Panobinostat (58) (See Figure 2). Dasatinib, a tyrosine kinase inhibitor with some antiviral properties, showed some promise as a HIV cure strategy when it was shown to reduce the viral reservoir of PWH with CML who were on simultaneous treatment with ART (60). Other senotherapeutics have been evaluated for potential geroprotective effects. One such drug examined in PWH is metformin, an anti-diabetic agent with anti-inflammatory properties. Historically used with caution in PWH due to risk of lactic acidosis and weight loss, recent studies in PWH have found that metformin benefits microbiota composition, promotes gut barrier integrity and reduces inflammation in diabetic and non-diabetic PWH(61,62). Nevertheless, to date, no clinical trials employing senotherapeutics to target age-related diseases have been undertaken in PWH. Encouragingly, a recent trial has been approved for development by the AIDS Clinical Trial Group (ACTG) to look at Dasatinib and Quercetin in PWH.

## The Kynurenine pathway as a senotherapeutic target

The KP has recently been identified as a promising target to increase healthy longevity in age-related diseases. Therapies targeting the KP so far have focused on cancer-related therapeutics through the use of IDO inhibitors, which weaken the immunosuppressive functions by suppressing the KP, starving the cell of energy (63). Therapeutic targets from a senotherapy perspective takes the opposite approach, increasing energy production. Given the role NAD+ has in the ageing process, efforts to increase NAD+ levels by increasing the availability of NAD+ have been key targets of research. Some studies have shown that raising intracellular NAD+ levels with NAD+ precursors, nicotinamide mononucleotide (NMN), and nicotinamide riboside (NR) may offer a promising therapeutic strategy for age-associated degenerative diseases in general and to extend lifespan. Achieving this goal is challenging due to inter-individual variations in NAD+ levels that are affected by age, gender, diet, exercise, genetic factors and individual health status (64).

CD38 is another attractive target as a therapy to treat NAD+ age-related metabolic dysfunction. CD38, a glycoprotein widely expressed in immune cell types, is involved in T-cell activation and can be used as a predictor of HIV disease progression, as well as being linked to monocyte activation and CVD outcomes in PWH (47,67). Functionally, CD38 is mainly a NADase, degrading circulating NAD and its precursor NMN, preventing NAD synthesis and regulating T-cell responses(68). Thus, increases in CD38 have been hypothesised as the main regulator of age-related NAD+ decline in ageing and metabolic disorders. Studies looking at CD38 inhibitors (CD38i) have shown anti-ageing properties of the thiazoloquin(az)olin(on)e 78c, reversing age-related NAD+ decline in mice. Other agents such as the CD38 monoclonal antibody daratumumab, have shown promising results in multiple myeloma patients (69).

As well as altering the mechanisms of ageing, targeting KP signalling networks, or its metabolic pathways harbors high potential to prevent and treat metabolic diseases. NMN was recently shown to improve insulin sensitivity in prediabetic women (70). In addition, a recently developed oral antidiabetic medication, imeglimin, can enhance glucose-stimulated ATP generation and induce the synthesis of NAD+ in pancreatic islets derived from diseased rodents with type 2 diabetes (71). Currently, there are over 15 clinical trials underway (72) that aim to assess the clinical efficacy of NAD+ precursors as a therapeutic strategy to attenuate markers for metabolic dysfunction. To date, however, none of these clinical trials are investigating the application of these therapies in PWH.

## Gaps in knowledge and future perspectives

Few therapeutic trials targeting the KP pathway or NAD+ precursors have looked specifically at HIV-related inflammation. However, one recent clinical trial investigated whether treatment with the neurokinin-1 receptor antagonist, aprepitant, alters tryptophan metabolism in PWH. They found that aprepitant decreased both kynurerine and tryptophan levels in ART-naive subjects but in PWH on ART, it caused a significant decreased the KTR (73). Targeting the KP has vast translational potential to target, prevent and treat age-related comorbidities in PWH and beyond. Additional studies will be needed to further delineate the role between the KP and the gut microbiome in PWH and whether the KP could be targeted by specific therapies to prevent or treat age-related comorbidities in PWH.

## Conclusion

With a growing aging population of PWH come new challenges with the management of age-related comorbidities. Understanding the link between gut dysfunction, inflammation, the KP and these comorbidities is important to not only optimise health outcomes, but also help predict those most at risk. Advances in Geroscience-based approaches and senotherapeutics offer a novel paradigm for addressing age-related disease in chronic HIV infection, refocusing the goals of successful ageing in PWH.

## Key points

- HIV infection is associated with persistent inflammation with translocation of microbes and/or microbial products from the gastrointestinal tract to the circulation; this process persists even with successful antiretroviral treatment.
- HIV infection is also associated with a reduction in diversity and alteration of the microbial composition with reduced abundances of beneficial bacteria and enrichment of potentially proinflammatory bacteria.
- The KP is involved in the control of immune responses and inflammation and has been associated with HIV disease progression and metabolic outcomes in PWH.
- Geroscience-based approaches, through the use of senotherapeutics could prove beneficial for improving health outcomes in age-related diseases seen in PWH.
- Treatment strategies targeting the KP pathway using NAD+ precursors and CD38ases could present a promising therapeutic target and further studies are needed in PWH.
